# Genome wide association analysis of sorghum mini core lines regarding anthracnose, downy mildew, and head smut

**DOI:** 10.1371/journal.pone.0216671

**Published:** 2019-05-14

**Authors:** Ezekiel Ahn, Zhenbin Hu, Ramasamy Perumal, Louis K. Prom, Gary Odvody, Hari D. Upadhyaya, Clint Magill

**Affiliations:** 1 Department of Plant Pathology & Microbiology, Texas A&M University, College Station, Texas, United States of America; 2 Department of Agronomy, Kansas State University, Manhattan, Kansas, United States of America; 3 Kansas State University, Agricultural Research Center, Hays, Kansas, United States of America; 4 USDA-ARS Southern Plains Agricultural Research Center, College Station, Texas, United States of America; 5 Texas A&M AgriLife Research, Corpus Christi, Texas, United States of America; 6 ICRISAT, Patancheru, Telangana, India; 7 King Abdulaziz University, Jeddah, Saudi Arabia; Bhabha Atomic Research Centre, INDIA

## Abstract

In previous studies, a sorghum mini core collection was scored over several years for response to *Colletotrichum sublineola*, *Peronosclerospora sorghi*, and *Sporisorium reilianum*, the causal agents of the disease anthracnose, downy mildew, and head smut, respectively. The screening results were combined with over 290,000 Single nucleotide polymorphic (SNP) loci from an updated version of a publicly available genotype by sequencing (GBS) dataset available for the mini core collection. GAPIT (Genome Association and Prediction Integrated Tool) R package was used to identify chromosomal locations that differ in disease response. When the top scoring SNPs were mapped to the most recent version of the published sorghum genome, in each case, a nearby and most often the closest annotated gene has precedence for a role in host defense.

## Introduction

Like all crops, sorghum (*Sorghum bicolor* (L.) Moench) is subject to attack by pathogens that cause yield loss and reduce grain quality in susceptible varieties. This makes identification of sources of disease resistance, whether single gene or multigenic, critical for successful breeding projects. Fortunately, there is a great deal of genome diversity in sorghum that can be used to counter virulence changes that occur in pathogen populations. Here we describe results of screening a large number of sorghum cultivars for resistance responses to three common pathogens as well as identification of genes that appear to be involved in resistance.

Among over 37,000 accessions of a sorghum germplasm collection at the International Crops Research Institute for the Semi-Arid Tropics (ICRISAT) gene bank, a core collection of 2247 accessions was developed in 2001, but this core collection was considered to be too large for many studies involving replicated evaluation. Hence, a sorghum mini core (10% accessions of the core or 1% of the entire collection) was developed from the existing core collection [1]. In order to maximize genetic diversity, it was developed by including all races of sorghum accessions collected from around the globe. The mini core collection has been included in several genotyping by sequencing (GBS) projects. SNP markers were first used with the mini core collection to identify prospective genes underlying traits such as plant height and maturity [2]. A GWAS analysis of the mini core collection was also conducted for drought tolerance traits [3]. Wang et al. used 13,390 SNPs to examine genetic structure and linkage disequilibrium in the mini core accessions [4]. As would be expected, the structure generally tracked the known races and geographic origins. Other than a large block (> 20 megabases) in the short arm of chromosome 6, smaller regions in the 10 kb range that showed evidence for selection appeared to be associated with genes for photosensitivity and grain/panicle architecture [4]. In a GWAS study very similar to this that examined resistance to anthracnose isolates from Pantacheru, India, 14,739 SNPs showed association with 8 loci, 7 of which were in regions that include genes previously associated with disease resistance responses [2]. In that study a cutoff *p* value of 10^−4^, somewhat below the Boniferri suggested cutoff was justified based on additional biologically relevant information [5]. More recently, the mini-core collection was included with other diverse accessions in a study of agroclimatic traits. That study, which also included linkage disequilibrium analysis and phylogenetic relationships took advantage of the locations of 265,487 Single Nucleotide Polymorphisms (SNPs) [6].

Anthracnose caused by the fungal pathogen *Colletotrichum sublineola* (or *sublineolum*) is one of the most devastating diseases in sorghum [7], and losses caused by the panicle phase of anthracnose in terms of grain yield are up to 30–50% [8]. Microsclerotia, seed transmission, and alternative hosts have been implicated as primary sources of inoculum [8]. The conidia germinate and develop appressoria and penetrate the epidermis directly or enter through stomata which is followed by an acervulus formation [8]. Whether measured by DNA–based tests or ability to infect different host cultivars, Colletotrichum is highly variable. In testing isolates collected from 6 populations from India using 15 host differentials, Thakur at al. [9] found differences in all six, as was also true for Random Amplified Polymorphic DNA (RAPD) Polymerase Chain Reaction (PCR) product electrophoretic patterns. Moore et al. [10] defined 13 pathotypes among 87 anthracnose isolates from Arkansas, including 11 when testing pathogenicity using 8 host differentials. Prom et al, found 17 different pathotypes on 10 host differentials from 235 US isolates that also differed in Amplified Fragment Length Polymorphism (AFLP) patterns [11]. The latter reference also pointed out that two lines resistant to all isolates had shown differential responses in an earlier study in Brazil.

Sorghum downy mildew, caused by *Peronosclerospora sorghi*, can create severe epidemics, resulting in heavy yield loss [12]. Oospores in the soil germinate by a germ tube and invade the roots of sorghum seedlings, and mycelium of the pathogen progress upward, colonizes the foliar meristematic tissues, and induces leaf chlorosis [8]. Conidia produced on the chlorotic leaves are disseminated to leaves of adjacent seedlings [8]. Since *P*. *sorghi* causes downy mildew in maize as well, it has been subdivided into 'sorghum/maize' and 'maize' infecting strains [12]. As for anthracnose, there are different pathotypes as defined by ability to infect host plants with different resistance genes, but in this case much less variation has been reported. Race 6 was detected in Texas in 2005 based on its occurrence on previously resistant commercial sorghum varieties [13] and was used exclusively in this study. No reports of new races have since appeared.

Head smut, caused by the soil-borne facultative biotrophic basidiomycete *Sporisorium reilianum* (Kühn) Langdon & Fullerton (syns. *Sphacelotheca reiliana* (Kühn) G.P. Clinton and *Sorosporium reilianum* (Kühn) McAlpine, is an important sorghum disease that has been reported from all parts of the world where sorghum is grown [14]. Soilborne spores germinate and penetrate the nodal region of the shoot apex [8]. It grows through the plant as a dikaryotic hypha formed between compatible mating types and is not generally detected until the time of heading when the fungal sorus replaces the sorghum inflorescence. At present 6 races have been defined in the US, but because of the use of different host differentials, it is not known if races defined elsewhere are unique.

In previous studies, the sorghum mini core collection was scored over several years for response to *C*. *sublineola*, *P*. *sorghi*, and *S*. *reilianum* [15] (S1 Table). In order to search potential candidate defense related genes against each of the three pathogens, the screening results of these three common sorghum diseases were combined with over 290,000 SNP loci from a recently updated version of a publicly available genotype by sequencing dataset available for the mini core collection [6]. Based on GWAS results, here we report top defense related candidate genes identified for each of these three pathogens.

## Materials and methods

### Disease screening and rating*s*

#### Anthracnose

Each of the 242 mini core accessions and three control cultivars were evaluated two times consecutively in September 2007 and January 2008 in the USDA-ARS green house, College Station, Texas as described in [16]. BTx635 (resistant) and B1 and BTx623 (susceptible) were included as checks in each experiment. The experiments were conducted under randomized block design replicated four times. A mixture of five anthracnose isolates which are aggressively virulent and commonly present in Texas was used as inoculum. The isolates are maintained at the USDA-ARS, Southern Plains Agricultural Research Center, College Station, Texas, USA. An effective spray inoculation technique and disease assessment method was used as previously described by Erpelding and Prom [17] and Prom et al. [16]. Briefly, inoculum for *C*. *sublineola* from each isolate was pooled following growth on ½ strength PDA plates. The spray inoculation was used at the 8–10 leaf stage (approx. 30 days after planting) to deposit approx. 3–5 ml conidial suspension (10^6^ conidia/mL) onto the leaves of each plant. Tween 20 (wetting agent) was added to the inoculum (0.5 ml/L). Immediately after spraying, plants were misted for 30 sec at 30–45 min intervals over a 10 hr/d period for one month. Disease assessments were conducted 30 days post-inoculation and thereafter, on a weekly basis for four weeks. Ratings were based on a scale of 1 to 5 [17], where 1 = no symptoms or chlorotic flecks on leaves; 2 = hypersensitive reaction (reddening or red spots) on inoculated leaves but no acervuli formation and no lesion development on other leaves; 3 = lesions on inoculated and bottom leaves with acervuli in the center; 4 = necrotic lesions with acervuli on inoculated leaves and infection spreading to bottom and middle leaves; and 5 = most leaves dead with abundant acervuli on the flag leaf. Accessions were considered resistant if plants in the row were rated as 1 or 2 and susceptible if rated as 3, 4, or 5. Thus the difference between a resistant and susceptible response as used here was the presence of acervuli on the leaves, which indicates successful reproduction of the pathogen.

#### Downy mildew

Inoculation of germinating seeds of 242 mini core accessions and subsequent scoring for downy mildew was detailed in Radwan et al. [15]. Each experiment was replicated three times using a randomized block design. In each replication, 16–17 inoculated seedlings were transplanted to a half-gallon pot, and allowed to grow in the greenhouse at 25° ± 1°C for 14 days. Each week, 15 to 20 accessions were tested at a time along with susceptible (Pioneer hybrid 84G62) and resistant (Pioneer hybrid 83P67) as checks. The sandwich inoculation technique was used as detailed by Thakur [18] for downy mildew resistance screening in the greenhouse. Spores were collected from plants systemically infected with virulent pathotype 6 being maintained year-round in the greenhouse on infected susceptible plants (Pioneer hybrid 84G62) and served as the source inoculum. As conidia production was found to be very low during the winter months, the greenhouse inoculation experiments were initiated in June 2009 and completed in May 2010 by skipping five months between October 2009 and February 2010. Susceptibility to downy mildew was evaluated two weeks after transplanting. Plants showing systemic and or local lesions were counted as infected. Disease incidence was determined from the percentage of infected plants in each replication and evaluated for disease symptoms. Accessions with 10% or less downy mildew incidence were considered resistant [19].

#### Head smut

All 242 mini core accessions along with BTx7078 (susceptible) and BTx635 (resistant) as checks were used for head smut resistance screening in the greenhouse. A set of fifty accessions along with two checks were planted in five-gallon pots starting April 2011 and at 15-day intervals the next 50 accessions and two checks were planted. Each experiment was conducted in a randomized block design with three replications. A five-gallon pot with five plants was maintained for inoculation in each replication. A reliable syringe inoculation technique as detailed by Perumal et al [20] was used for green house evaluation. Briefly, sporida were grown from teliospores that had been collected from infected sorghum plants in south and central Texas. Cultures were grown on a rotator in liquid culture (PDB) at 26° for 3 days and mixed for hypodermic inoculation. In susceptible interactions, the inflorescence is replaced with sori visible at the time of flowering, the time at which scoring was done. To verify resistance, all apparently healthy inflorescences were cut back to verify that tillers were not infected as occurs with systemic head smut infections. Since 37 photoperiod-sensitive lines did not flower, their main tillers were cut and the plants maintained until day length reduction induced flowering (mid November). Lines with no infection detected in any replicate were scored as resistant.

#### GWAS and SNP mapping

The SNPs data was extracted from an integrated sorghum SNPs dataset based on sorghum reference genome version 3.1.1 and originally genotyped using GBS [2,4,21,22]. The missing data were imputed using Beagle 4.1 [23]. GWAS was run using a linear mixed model in GAPIT with Model.selection = T, SNP.MAF = 0.01 [24,25]. The Manhattan plots were made using qqman package [26]. SNPs with high probability of contribution to each of the three diseases responses were tracked to the specific chromosome location based on the sorghum reference genome sequence, version 3.1.1 available at the Phytozome 12 (https://phytozome.jgi.doe.gov), updated in 2018 [27].

## Results

As part of a study supported by the Global Crop Diversity Trust, the sorghum mini core lines were examined for response to *Colletotrichum sublineola* with a mix of spores from isolates causing disease in Texas. They were also specifically examined for response to race 6 of *P*. *sorghi* and to a mix of *S*. *reilianum* isolates collected from several locations in Texas. For anthracnose, 123 of the 245 lines that could be scored were resistant, for downy mildew 52 of the 240 mini core lines tested were resistant and for head smut, 102 of 229 lines were classed as resistant. (S1 Table)

All together 459,304 SNPs went through filtering process and generated 299,204 SNPs for anthracnose, 306,615 for downy mildew, and 290,299 for head smut. Because of the very high number of comparisons possible, a very high cutoff score is generally employed. However, since there is also a possibility that differential responses to races of the pathogens and that single resistance genes may not be detected if not present in enough accessions of the extremely diverse mini core collection to be detected, we opted to examine the highest scoring SNPs for each pathogen in order to determine if they identified genes known to be involved with host defense responses or had been identified in other disease association studies. The results also show the distance in base pairs to the nearest genes or physically nearby genes with defined functions.

### Anthracnose

**Fig 1:**
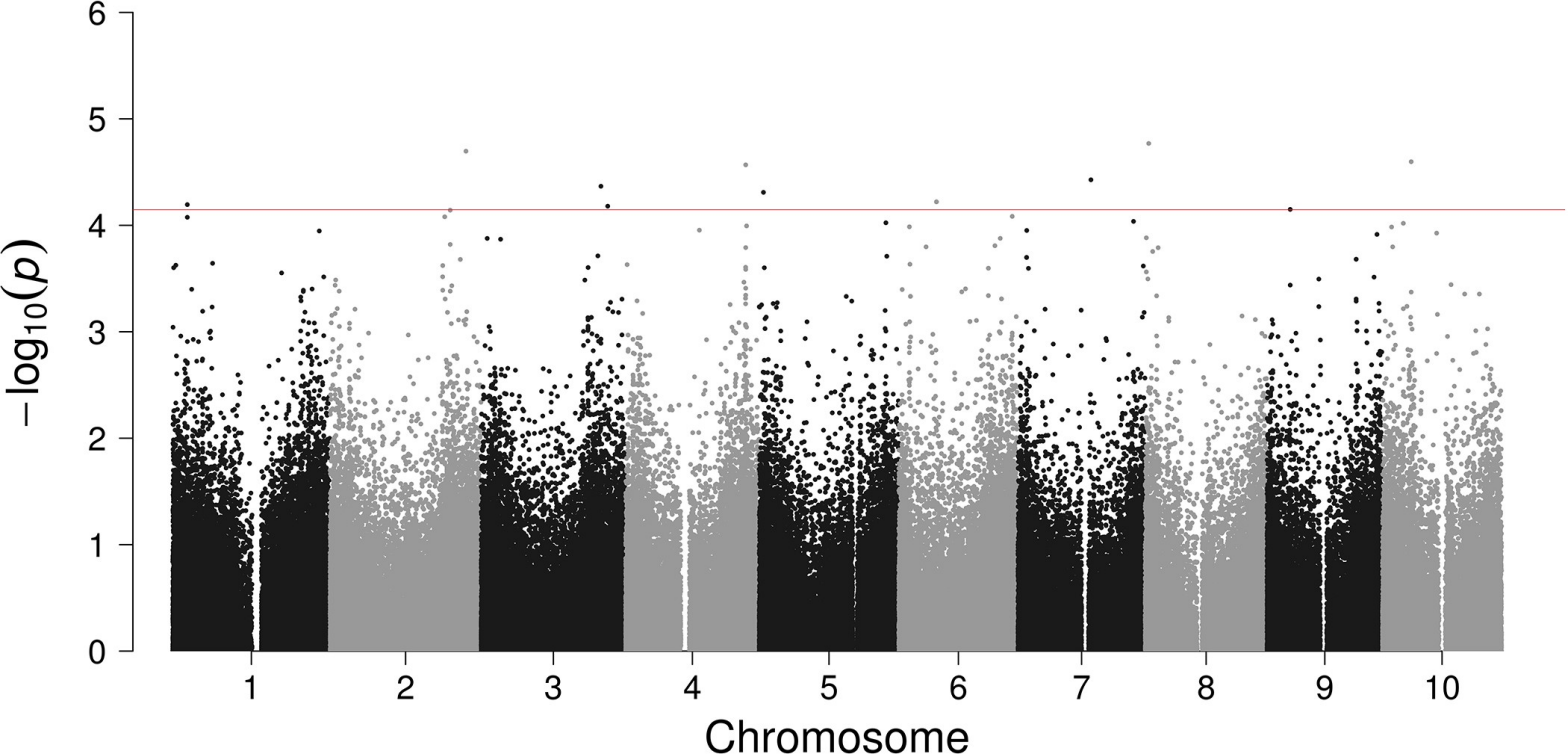
Genome-wide association analysis for anthracnose in sorghum mini core collection. Manhattan plot demonstrates the top candidate SNPs. The line is a cut-off for top candidate genes listed in Table 1.

Summary data for anthracnose are show in Fig 1 and Table 1. The highest probability for a SNP associated with resistance/susceptibility to anthracnose is associated with a Zinc-finger-homeodomain (ZF-HD) protein encoded by a gene on chromosome 8. ZF-HD proteins have known functions in plant defense through activation of calmodulin isoform 4 (*GmCaM4*) gene expression in soybean [28]. Next is SNP S02_69955660 on chromosome 2, that is 5134 bases from an F-box domain coding region. F-box proteins are involved in cell death and defense responses in tobacco, tomato [29], and Arabidopsis [30]. Cuevas et al. also reported that an F-box protein is one of the top candidate genes related to sorghum defense response against *C*. *sublineola i*n a GWAS using a different set of sorghum cultivars [31].

**Table 1:** Top SNPs associated with anthracnose.

Chr.	Location	Nearest gene and function^a^	Base pairs away	P value
8	1802680	Sobic.008G020700 Similar to Zinc finger homeodomain (ZF-HD) protein dimerisation region containing protein	2605	0.000017
2	69955660	Sobic.002G330900 Predicted protein. F-box domain.	5134	0.000020
4	61746687	Sobic.004G273600 Similar to H0215F08.15 protein. RNA recognition motif. (a.k.a. RRM, RBD, or RNP domain). Exodeoxyribonuclease VII.	8004	0.000027
7	37557648	Sobic.007G105400 Similar to Ubiquitin carrier protein. Ubiquitin-conjugating enzyme (E2)	28912	0.000037
3	61650258	Sobic.003G281500 Similar to Pleiotropic drug resistance protein 15. ABC transporter domain, ABC-2 type transporter domain, Plant PDR ABC transporter associated protein domain, and ABC-transporter extracellular N-terminal domain containing	5220	0.000043
5	2211068	Sobic.005G024400 Ribosome Assembly protein WD domain, G-beta repeat, Histone-binding protein RBBP4 or subunit C of CAF1 complex.	0	0.000049
6	19369682	Sobic.006G040101	54956	0.000060
	19369687	Plant transposase (Ptta/En/Spm family)		
1	7499623	Sobic.001G097400 Similar to Phosphatidylinositol-3-and 4-kinase family protein, expressed. WD domain, G-beta repeat containing	268	0.000064
3	65141341	Sobic.003G325100 Similar to Blight resistance protein RGA3-like. NB-ARC domain and leucine rich repeat containing	7468	0.000066
9	11921711	Sobic.009G082200 Similar to Putative serine/threonine kinase protein.	26295	0.000071

^a^Top SNPs associated with anthracnose are listed. The tables also show the distance in base pairs to the nearest genes with functions.

A number of plant RNA-binding proteins (RBPs) have known roles in plant immune response regulation [32]. The nearest gene coding region of SNP S04_61746687 on chromosome 4 includes an RNA recognition motif. Moreover, a peroxidase related gene coding region is only 23,915 bps away from the same SNP. Among the proteins induced during host plant defense, class III plant peroxidases are well known [33].

According to Zhou et al., ubiquitin-conjugating enzymes as detected by the SNP on chromosome 7 play an essential role in both positive and negative plant responses to pathogens [34]. ABC transporters such as the gene nearest this SNP have been shown to be required for organ growth, plant nutrition, plant development, response to abiotic stresses, and pathogen resistance [35].

Plant ribosomal proteins are known to play a role in non-host disease resistance against bacterial pathogens in *Nicotiana benthamiana* [36]. In addition, a WD40 repeat is reported to be involved in cell wall formation in plants [37]. The SNP at S05_2211068 on chromosome 5 is within the coding region of a gene that may serve either or both of these functions. Also, this SNP is also only 6148 bps away from a leucine-rich-repeat protein coding region which is a common feature of known resistance genes.

Transposable elements are known to be able to affect plant gene expression and reduce host defense mechanisms [38]. The nearest annotated coding region of the two SNPs S06_19369682 and S06_19369687 on chromosome 6 is relatively near a region with a transposase signature.

Salicylic acid (SA) has a central role in defense against pathogen attack, and phosphatidylinositol 4-kinase activation is an early response to SA in Arabidopsis [39]. The SNP at S01_7499623 on chromosome 1 is 268 bps from a coding region which contains a member of the phosphatidylinositol-3-and 4-kinase family and a WD domain.

The majority of disease resistance genes in plants encode nucleotide-binding site leucine-rich repeat (NBS-LRR) proteins [40]. The SNP S03_65141341 on chromosome 3 is located close to the coding region similar to resistance gene analog RGA3, a member of the nucleotide-binding site (NBS)-leucine-rich repeat (LRR) gene [41]. The SNP on chromosome 9 is comparably close to a coding region of putative serine/threonine kinase, enzymes that are key to signal transduction. Receptor-like kinases (RLKs) are involved in a diverse array of plant responses including development, growth, hormone perception and the response to pathogens [42].

### Downy mildew

**Fig 2:**
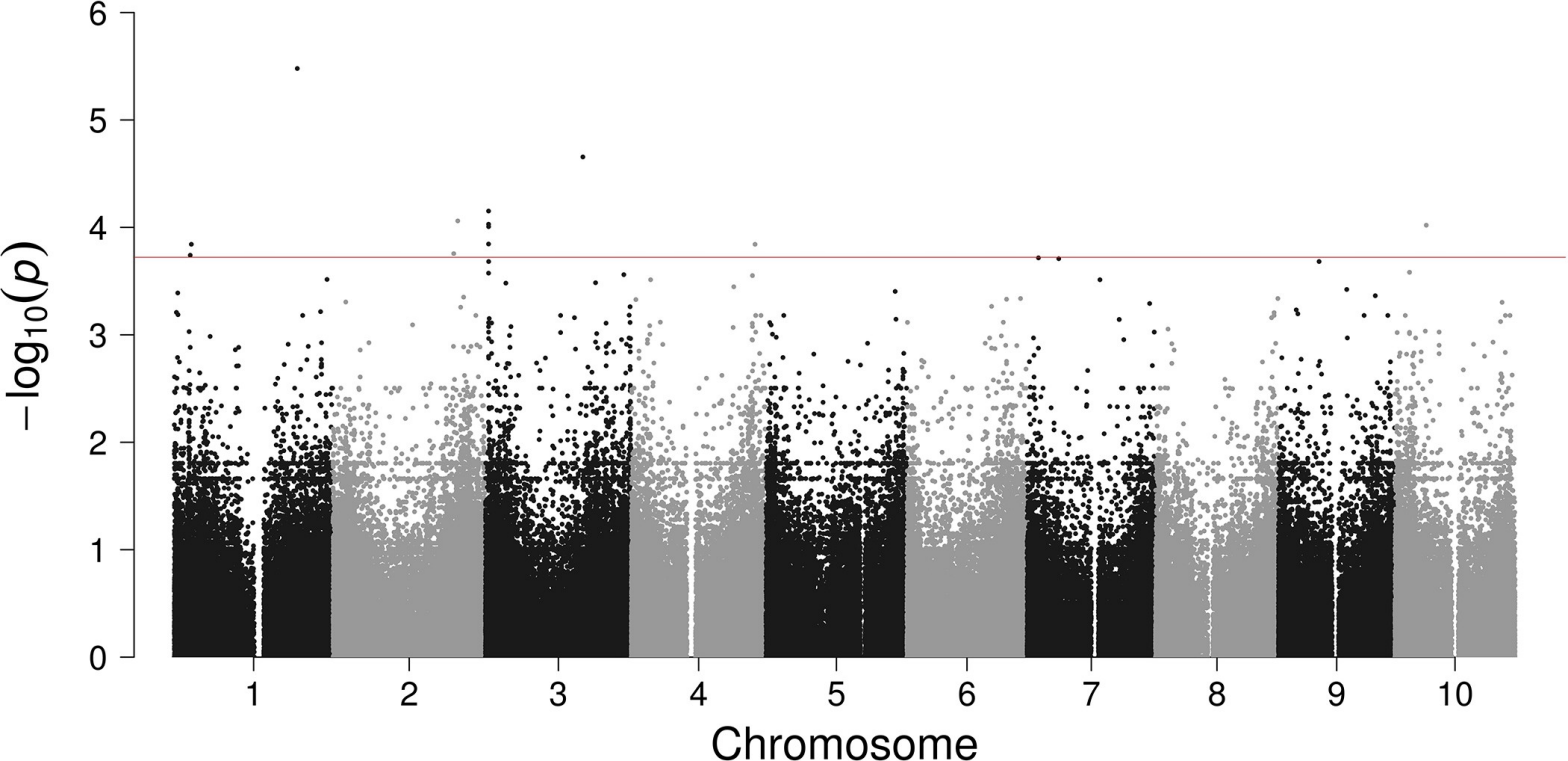
Genome-wide association analysis for downy mildew in sorghum mini core collection. Manhattan plot demonstrates the top candidate SNPs. The line is a cut-off for top candidate genes listed in Table 2.

Summary data for downy mildew are show in Fig 2 and Table 2. Glucose-6-phosphate dehydrogenase (G6PDH) plays a role in response to abiotic stresses and pathogenesis [43]. On chromosome 1, the SNP, located in 62708122, is statistically the most distinguishable with the lowest p-value.

**Table 2:** Top SNPs associated with downy mildew.

Chr.	Location	Nearest/nearby genes and function^a^	Base pairs away	P value
1	62708122	Sobic.001G339100 Similar to Glucose-6-phosphate 1-dehydrogenase, pentose phosphate pathway (oxidative branch)	254	0.0000033
3	49693492	Sobic.003G185000 PF05266—Protein of unknown function (DUF724) (DUF724), Agenet domain containing protein.	15551	0.000022
3	1603221	Sobic.003G018300	22460*	0.000070
	1608048	Similar to Os01g0213000 protein Ring finger	17633	
	1608046	domain containing protein	17635	
2	63642528	Sobic.002G249000 Similar to AT-hook motif nuclear localized protein 2.	7612*	0.000087
10	15963823	Sobic.010G126500 Aspartyl proteases Xylanase inhibitor N-terminal and C-terminal containing protein	54532	0.000095
3	1636714	Sobic.003G018600 Similar to Small nuclear ribonucleoprotein homolog	0	0.000098
1	8688112	Sobic.001G111300 Uncharacterized protein Protein tyrosine kinase.	4318*	0.000144
4	63086639	Sobic.004G289700 Predicted Ca2+-dependent phospholipid-binding protein C2 domain containing protein	11853*	0.000144
2	61590648	Sobic.002G224300 Predicted protein No apical meristem (NAM) protein containing.	0	0.000175
1	8131244	Sobic.001G105900 Similar to Acc synthase ethylene biosynthesis I pathway Aminotransferase class I and II.	10046	0.000182

^a^Top SNPs associated with downy mildew are listed. The tables also show the distance in base pairs to the nearest or nearby genes (*) with functions.

* = Not nearest, but nearby genes

The *Arabidopsis thaliana* gene *enhanced downy mildew 2* (*EDM2*) encodes a nuclear protein required for RPP7-mediated race-specific disease resistance against *Hyaloperonospora arabidopsidis*, proper floral transition and additional developmental processes [44]. By yeast two-hybrid screening for EDM2-interacting proteins, Tsuchiya & Eulgem identified AtEML1, a member of a small group of four Arabidopsis proteins containing an EMSY N-terminal domain, a central Agenet domain, and a C-terminal coiled-coil motif [44]. This suggests the Agenet domain containing protein on chromosome 3 could play a similar role in sorghum.

RING finger proteins comprise a large family and play important roles in regulation of growth and development, hormone signaling, and responses to biotic and abiotic stresses in plants [45]. Among the list of top candidates, 3 SNPs on chromosome 3 are all closest to a RING finger domain protein.

It is reported that AHL20, an AT-hook containing DNA-binding protein, negatively regulates pathogen triggered immunity (PTI) [46] in Arabidopsis, and the SNP S02_63642528 on chromosome 2 near a nuclear localized, AT-hook-motif containing protein.

The SNP S10_15963823 on chromosome 10 is near a coding region that contains both aspartyl protease and xylanase inhibitor activity in the N- and C-termini. Aspartyl protease-mediated cleavage of Bcl-2-associated anti-apoptosis gene product of (BAG)6 is necessary for autophagy and fungal resistance in plants [47]. Further, xylanase inhibitor proteins (XIP) are potential defense molecules, which could act to prevent plant cell wall degradation by fungal hydrolytic enzymes [48].

Alternative splicing (AS) functions in a range of physiological processes, including plant disease resistance [49]. The SNP S03_1636714 on chromosome 3 is located on the coding region of a protein similar to small nuclear ribonucleoprotein which is highly involved in AS. Moreover, the SNP is only 5607 bp away from a ring-finger domain protein.

Plant receptor protein kinases (RPKs) represent the main plasma membrane pattern recognition receptors (PRRs) that can detect diverse microbe-associated molecular patterns (MAMPs) [50].

In a GWAS study with another sorghum collection, Cuevas et al reported a tyrosine-kinase as one of top candidate resistance genes for sorghum against *C*. *sublineola* [31]. The SNP S01_8688112 on chromosome 1 is only 4318 bp away from a tyrosine-kinase coding region.

In Arabidopsis, the C2 domain protein BAP1 negatively regulates defense responses [51]. Similarly, on chromosome 4, we found the SNP S04_63086639 near the coding region of a C2 domain.

NAC (NAM, ATAF1&2, and CUC2) genes play roles in plant growth and development ranging from the formation of shoot apical meristem, floral organ development, reproduction, lateral shoot development, and defense responses to biotic and abiotic stresses [52]. The SNP S02_61590648 on chromosome 2 is in the coding region with homology to an apical mannose binding lectin coding region from pepper. Plant mannose-binding lectins (MBLs) are crucial for plant defense signaling during pathogen attack by recognizing specific carbohydrates on pathogen surfaces [53].

One of the earliest detectable events during plant-pathogen interaction is a rapid increase in ethylene biosynthesis [54]. It is also known that aminotransferases confer enzymatic resistance to downy mildew in melon [55]. The SNP S01_8131244 on chromosome 1 is nearby a coding region similar to ACC synthase, which is related to ethylene biosynthesis I pathway, and aminotransferase class I and II domains.

### Head smut

**Fig 3:**
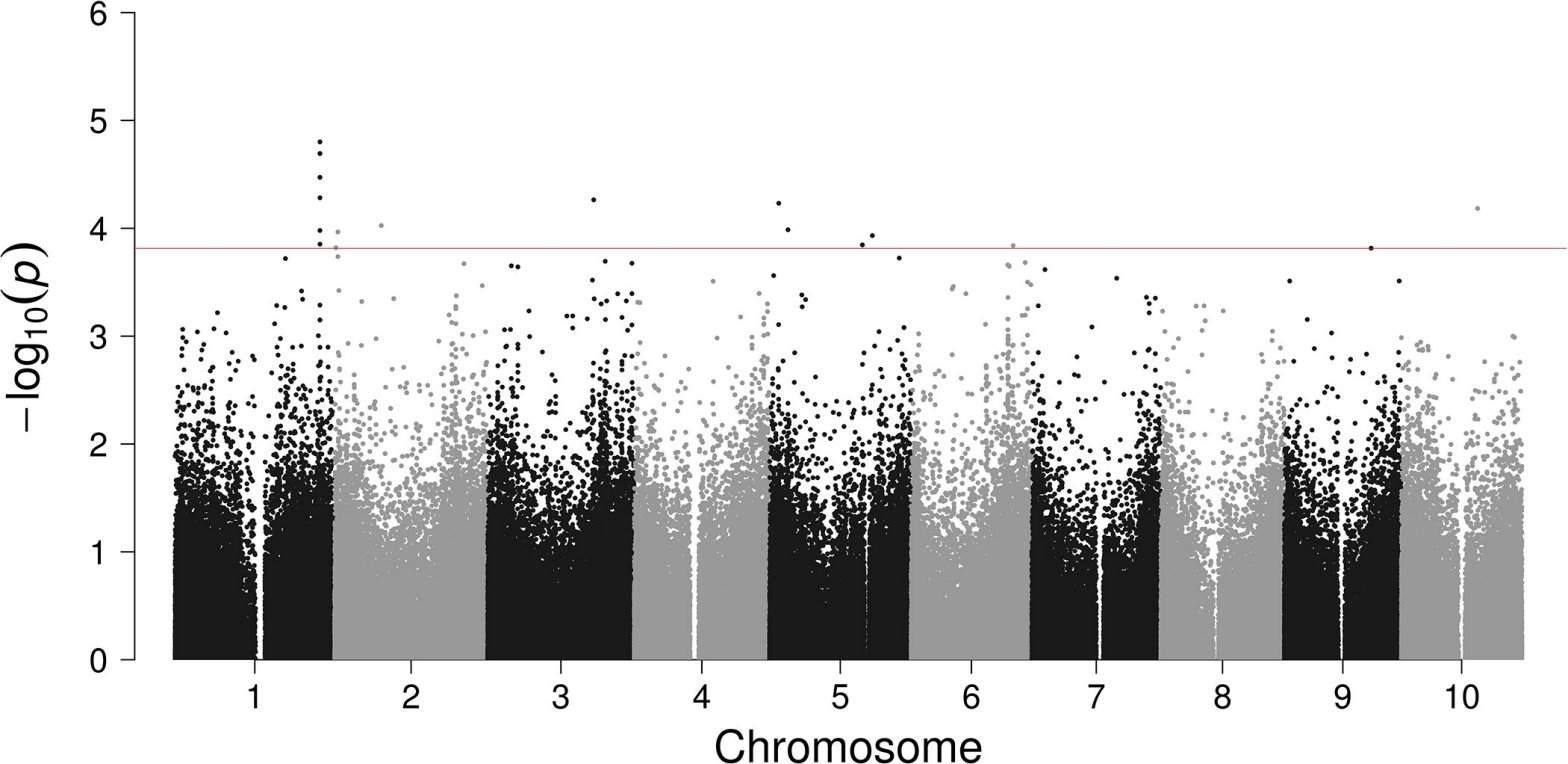
Genome-wide association analysis for head smut in sorghum mini core collection. Manhattan plot demonstrates the top candidate SNPs. The line is a cut-off for top candidate genes listed in Table 3.

Summary data for headsmut are shown in Fig 3 and Table 3. As previously mentioned most plant and animal immune receptors have a leucine-rich repeat (LRR) domain [56], and LRR proteins are known to take a significant role in plant defenses [57]. Three SNP Manhattan plot peaks were associated with LRR protein encoding genes, two on chromosome 1 and one on chromosome 5. In sorghum NB-LRR resistance genes are found in clusters on several chromosomes, including chromosomes 1 and 5 [58].

**Table 3:** Top SNPs associated with head smut.

Chr.	Location	Nearest gene and function^a^	Base pairs away	P value
1	73516778 and 7 more within 2000 bp	Sobic.001G459500 Similar to Leucine Rich Repeat family protein, expressed.	271	0.000016
3	53833202	Sobic.003G207500 Minor histocompatibility antigen H13 Signal peptide peptidase	25606	0.000054
5	4712627	Sobic.005G049600 Weakly similar to H0607F01.6 protein Protein tyrosine kinase	0	0.000059
10	38702488	Sobic.010G143900 Similar to Os06g0470000 protein Glycosyltransferase	85520	0.000066
2	23750579	Sobic.002G142900 Similar to Putative uncharacterized protein, Tetratricopeptide repeat-containing protein	5417	0.000094
5	9400668	Sobic.005G076301 Coiled-coil domain containing protein lobo homolog Leucine-rich repeat containing protein	0	0.000103
2	1751971	Sobic.002G019000 Similar to Putative uncharacterized protein, Xyloglucan fucosyltransferase containing.	10608	0.000108
5	52227160	Sobic.005G120201 Glutathione S-transferase	1426	0.000117
1	73523579	Sobic.001G459600 Weakly similar to Putative uncharacterized protein Leucine Rich Repeat containing protein	0	0.00014
6	51844045	Sobic.006G160500 Similar to OSIGBa0159I10.13 protein, Aspartyl proteases Xylanase inhibitor N-terminal containing	0	0.000145
2	734336	Sobic.002G007800 Similar to Os05g0302300 protein Zinc finger containing protein	0	0.000151

^a^Top SNPs associated with head smut are listed. The tables also show the distance in base pairs to the nearest genes with functions.

Signal peptide peptidase (SPP) plays a crucial role in life processes including immunological response in vertebrates, and SPPs are found in plants [59]. On chromosome 3, SNP S03_53833202 is located near a signal peptide peptidase gene. Since these genes are critical for secreting or delivering proteins to correct membrane locations, a role in defense is logical, though not proven.

Plant receptor protein kinases (RPKs) represent the main plasma membrane pattern recognition receptors (PRRs) that function in perceiving diverse microbe-associated molecular patterns (MAMPs) [60]. As for downy mildew, but on a different chromosome a SNP was detected in a tyrosine-kinase gene on chromosome 5.

Glycosyltransferases of plant secondary metabolism transfer nucleotide-diphosphate-activated sugars to low molecular weight substrates, and, additionally, it has been suggested that glycosyltransferases have an important role in plant defense and stress tolerance [61]. A SNP on chromosome 10 is near a glycosyltransferase coding region.

It is known that one of the tetratricopeptide repeat (TPR) proteins known as SRFR1 (suppressor of rps4-RLD 1) functions negatively in resistance toward the effector molecule for AvrRps4 in Arabidopsis [62]. It seems likely TPR proteins also affect resistance in sorghum since a SNP nearby a TPR coding region on chromosome 2 was detected.

Cell walls are crucial for disease resistance in plants, and xyloglucan fucosyl transferase is a well-known enzyme involved in plant cell wall biosynthesis [63]. On chromosome 2, a SNP near a xyloglucan fucosyl transferase coding region was found.

Glutathione (GSH) is a non-protein thiol compound that has been repeatedly reported to play an important role in plant responses during biotic stresses [64]. A SNP was detected very near a glutathione S-transferase.

As described earlier, aspartyl protease-mediated cleavage of BAG6 is necessary for autophagy and fungal resistance in plants [47]. Xylanase inhibitor proteins (XIP) are potential defense molecules, which could act to prevent plant cell wall degradation by fungal hydrolytic enzymes [48]. The SNP detected within the coding region of aspartyl proteases and XIP N-terminal on chromosome 6 is congruent with previous studies.

Cuevas et al. reported LRR, tyrosine-kinase, and zinc finger proteins are top candidate resistance genes for sorghum against *C*. *sublineola* [31]. As mentioned previously, three SNPs were found that are near or within LRR tyrosine-kinases were found. Additionally, zinc finger protein, which plays essential roles in plant responses to biotic and abiotic stress, was directly tagged by a SNP on chromosome 2 [65]. The results strongly suggest that LRR, tyrosine-kinase, and zinc finger proteins are involved in sorghum immunity against both the facultative biotrophs *C*. *sublineola* and *S*. *reilianum*, and possibly other pathogens as well.

## Discussion

Sorghum is divided into five races (bicolor, caudatum, guinea, durra, kafir), along with the ten intermediate races resulting from all possible inter-race crosses [66]. With the globally available sorghum germplasm (~40,000), potentially 25% the germplasm has been exploited so far through different breeding programs to improve yield potential. The remaining 75% is still under-exploited, partly due to the photo-sensitive nature of many lines, as well as many un-adapted agronomic traits. The mini core represents geographically diverse germplasm sources in sorghum covering both photo-period sensitive and insensitive accessions. In this study we identified nine photo-sensitive: IS7305 (Nigeria), IS9745 (Sudan), IS13549 (Mexico), IS16528 and IS30572 (Cameroon, Central Africa), IS20632 (USA), IS29239 (Eswatini, South Africa), IS31557 (Burundi, East Africa)) and IS31714 (Yemen) and four photo insensitive: IS2413 (Iran), IS26749 (South Africa), IS29358 and IS29392 (Lesotho, South Africa) germplasm accessions as multiple sources for resistance to anthracnose, SDM and head smut (S1 Table). These nine photo-sensitive potential sources are currently in the US sorghum conversion program using BTx406 as the female parent. The other four photo-insensitive sources are being used in other breeding programs as potential sources. The mini core collection represents the core collection of sorghum, which can be evaluated extensively for agronomic traits including resistance to biotic and abiotic stresses to identify accessions with desirable characteristics for use in crop improvement research and genomic studies [1]. With reduced number, but including core components of sorghum accessions, mini core collection surely utilizes research by reducing labor intensity and cost of money. This GWAS study took advantage of convenience provided by mini core collection.

In this research, we took advantage of disease rating data for up to 242 accessions from the minicore collection and the availability of a publicly available genome sequencing project for those same accessions to identify SNPs that may be associated with resistance. A similar study made earlier [2] used sequenced GWAS to identify SNPs associated with anthracnose resistance. In that study 14,739 SNPS identified 8 regions on chromosomes 1, 6, 8 and 10 which could be associated with potential disease-related genes at a p value of 10^−4^ or lower. The genes identified ranged from being 23 bp to 49 kb from the identifying SNP. Here, with the whole genome sequences available, over 290,000 useful SNPS were available for association mapping with anthracnose, so only genes that are essentially adjacent to individual SNPs at a p value of 7×10^−5^ or less were examined for possible roles in disease. Putative host defense genes were found on chromosomes 1, 6 and 8, but they did not appear to be the same genes identified in the earlier study. Here, genes on chromosomes 3, 5, 7 and 9 were also detected. In all but one of the SNPs examined, nearby genes have previously been implicated in disease responses in sorghum or other plants. Other QTLs mapped to chromosomes for anthracnose resistance include three genes on chromosome 6 and one on chromosome 4 [67]. Again, the locations do not match those revealed in this study. A major gene (or small gene cluster) on chromosome 5 from SC745-8 between 59.7 and 60.77 was not detected, suggesting it was either not present or present in too few mini-core accessions to be scored. The same is true for two classic R genes on chromosome 9 identified via differential expression [68].

Some top candidate SNPs were distant from the coding region of the nearest defense related gene. While GWAS identify many disease-associated SNPs, using them to decipher disease mechanisms is hindered by the difficulty in mapping SNPs to genes, and a recent study found that affected genes are often up to 2 Mbps away from the associated SNP, and are not necessarily the closest genes to the SNP [69]. This may be due to the role of chromatin remodeling in regulating transcription in eukaryotic organisms. Response elements that affect transcription in plants may be megabases away from the actual start site [70].

There are several factors that may explain the differences in the studies. First, our analysis was made simply on the basis of resistance (no fungal reproduction, even when lesions are present) or susceptibility rather than the 1–9 scoring system used in the earlier study. In addition, *C*. *sublineola* is an extremely variable pathogen that typically is controlled by single gene resistance via recognition of pathogen avirulence factors that trigger host recognition involving NB-LRR proteins. As a consequence, the isolates collected from Texas may differ significantly from those in the India study. Also, a few substitutions have been made to replace lines included in the original mini core accessions for reasons such as low seed supply. Here, a more recent update of the annotated sorghum genome sequence was used, and while the update resulted in some re-numbering, generally those changes are not great and none would change chromosomal assignments. Since all the genes identified in both studies have potential roles in host defense, all are deserving of additional analysis. Specifically, some top candidate SNPs, such as the SNP S01_73516778, are extremely close to known host defense related genes. It is important to pay attention to these SNPs, and further experiments, such as Real-Time qRT-PCR to measure gene expressions, is essential.

This is the first GWAS analysis of the mini core collection for downy mildew in sorghum. While quantitative trait loci (QTLs) for sorghum downy mildew and for sorghum head smut have been identified in maize, the syntenic relationships for the two species have not been resolved to the point of providing transferable information. In this case, only a single, newly discovered race 6 of the pathogen was used to inoculate plants of each accession. Over 306,000 useable SNPS were detected in the 240 mini core accessions successfully screened, of which 52 were resistant. As was the case with anthracnose the 10 most likely candidates (p < 1.8 × 10^−4^) were very near genes with functions predicting a potential role in host defense. In an earlier study, using 14,739 SNP markers, Upadhyaya et al 2013 have mapped eight loci linked to anthracnose resistance in through association analysis of the sorghum mini core collection evaluated for anthracnose resistance for 2 years in the field [71].

This is also the first case of using GWAS for head smut. Since symptoms can be scored only after heading, many of the lines could only be scored months after planting and inoculation. Here, over 290,000 SNPs could be used for the 229 accessions scored, of which 102 were resistant. Again, only R vs S responses were recorded but now R meant less than 10% of inoculated plants developed symptoms in the primary shoots or tillers.

Unless individual R genes providing race-specific resistance were present in a number of accessions it is unlikely they would be detected by this type of analysis. Especially in the case of anthracnose, where many races have evolved to overcome LRR type R genes, plants have also evolved a large family of such genes. The plasticity of NBS-LRR resistance genes in sorghum is driven by multiple evolutionary processes [72]. In fact, defense response genes typically occur in families with numerous copies [58] so it is not surprising those identified differ in location.

Overall, most of the genes identified are involved in aspects of host defense that would be typical of QTLs with minor effects rather than major genes. Those expected to be more directly involved in host defense, include SNPs near regions encoding zinc finger and LRR related proteins. Both were on the top list for anthracnose and head smut and near the top of the list for downy mildew. Tyrosine kinase related SNPs were on the top lists of downy mildew and head smut, but not on the top list of anthracnose. If expression of these genes can be verified to differ in resistant versus susceptible cultivars, allele specific differences will provide ideal molecular markers to speed resistance breeding. While tags associated with major genes would be directly useful for marker assisted selection for specific pathotypes, the ability to simultaneously screen for the presence of combinations of QTLs could lead to a more durable form of resistance.

## Supporting information

S1 Table Sorghum mini core germplasm -ICRISAT—Green house screening of Anthracnose (2008 & 2009), Head smut and Downy mildew (2009 & 2010). (DOCX)
