# Supplementary material for: Genome wide association analysis of sorghum mini core lines regarding anthracnose, downy mildew, and head smut
Source: PLoS One. 2019 May 14;14(5):e0216671. doi: 10.1371/journal.pone.0216671 (PMC6516728; doi:10.1371/journal.pone.0216671)
Supplement: S1 Table — (DOCX) [file pone.0216671.s001.docx]

| \| **S1 Table Sorghum minicore germplasm -ICRISAT - Green house screening of Anthracnose (2008 & 2009), Head smut and Downy mildew (2009 & 2010)** \| \| \| \| \| \| \| \| --- \| --- \| --- \| --- \| --- \| --- \| --- \| \|  \|  \|  \|  \| Disease \| \| \| \| **Collect. #** \| **IS number** \| **Alternate accession identifier** \| **country** \| **Anthrac- nose** \| **Head smut** \| **Downy mildew** \| \| FR 1 \| 608 \| FC13575; Ames amber \| USA \| S \| S \| S \| \| FR 2 \| 995 \|  \| USA \| R \| S \| S \| \| FR 3* \| 1041 \| PJ 7R; IC 3535; PI 246716 \| IND \| S \| S \| S \| \| FR 4 \| 1212 \| AS 5192; Chu-yeh-ching \| CHN \| S \| S \| R \| \| FR 5 \| 1219 \| AS 5224 \| CHN \| S \| S \| S \| \| FR 6 \| 1233 \| Kaolaing \| CHN \| R \| S \| S \| \| FR 7 \| 2205 \| IC 6183; Jaglur \| IND \| S \| S \| S \| \| FR 8 \| 2382 \| P 3736; Q2-5-44; PI 229841 \| ZAF \| R \| S \| S \| \| FR 9 \| 2389 \| Q2-5-73; PI 229865; Mimosa park \| ZAF \| R \| R \| S \| \| FR 10 \| 2397 \| Q2-5-81; PI 229874; Red kafir corn \| ZAF \| R \| R \| S \| \| FR 11 \| 2413 \| PI 222835; Vulgare astaraazerbaijan \| IRN \| R \| R \| R \| \| FR 12 \| 2426 \| Q2-3-58; PI 220636 \| AFG \| S \| S \| S \| \| FR 13 \| 2864 \| Var. red hegari DL/59/1541 \| ZAF \| S \| R \| S \| \| FR 14 \| 2872 \| Glza 5/59; FAO 8470 \| EGY \| S \| S \| S \| \| FR 15* \| 2902 \| Baruoam doke \| NGA \| R \| S \| S \| \| FR 16* \| 3121 \| PI 226096 \| KEN \| S \| S \| R \| \| FR 17 \| 3158 \| DL 60/122; Early goloa \| ZAF \| R \| S \| S \| \| FR 18 \| 3946 \| Sweet Sudan 59-3 \| IND \| R \| S \| S \| \| FR 19 \| 3971 \| IC 8946 \| IND \| R \| S \| S \| \| FR 20 \| 4060 \| Dudabli \| IND \| R \| S \| R \| \| FR 21* \| 4360 \| Chaklabhan \| IND \| S \| S \| R \| \| FR 22* \| 4372 \| Bandela white barkherahat \| IND \| S \| S \| R \| \| FR 23 \| 4515 \| Chetapuri bhendala \| IND \| S \| S \| S \| \| FR 24 \| 4613 \| Waradi udgir \| IND \| S \| S \| R \| \| FR 25 \| 4631 \| Gulbhendi khargaon \| IND \| S \| S \| R \| \| FR 26 \| 4698 \| Shallu hdir \| IND \| S \| S \| S \| \| FR 27 \| 5094 \| Saner \| IND \| S \| S \| R \| \| FR 28 \| 5386 \| Makkatai cholam \| IND \| S \| S \| S \| \| FR 29* \| 5529 \| Kesari padda jola \| IND \| S \| S \| S \| \| FR 30 \| 5667 \| Allu or alluna jola \| IND \| S \| S \| S \| \| FR 31* \| 5919 \| Shusowa \| IND \| S \| S \| S \| \| FR 32* \| 5972 \| Bedra khama \| IND \| S \| S \| S \| \| FR 33* \| 5999 \| Pritipur \| IND \| S \| S \| - \| \| FR 34* \| 6351 \| Jola Nandyal \| IND \| S \| S \| S \| \| FR 35* \| 6354 \| Jola Nandyal \| IND \| S \| S \| S \| \| FR 36* \| 6421 \| Jowar variety shenoli 4-5 \| IND \| S \| S \| S \| \| FR 37* \| 7305 \| BO 3; Shambul \| NGA \| R \| R \| R \| \| FR 38* \| 7310 \| BO 8; Fara fara \| NGA \| S \| R \| S \| \| FR 39* \| 7679 \| NG 112; Wyibuyi \| NGA \| R \| R \| S \| \| FR 40 \| 7987 \| SO 93; Dawa da \| NGA \| S \| - \| S \| \| FR 41 \| 8012 \| Kacosima \| JPN \| S \| S \| S \| \| FR 42 \| 8348 \| Red jaunpur (grain type) \| PAK \| S \| S \| S \| \| FR 43 \| 8774 \| E 265; S. basutorum \| ZAF \| S \| S \| S \| \| FR 44 \| 8777 \| E 268; Kabii 11 \| UGA \| S \| S \| S \| \| FR 45* \| 8916 \| E 581 \| UGA \| R \| R \| S \| \| FR 46* \| 9108 \| E 778 \| KEN \| S \| - \| S \| \| FR 47* \| 9177 \| E 1085; Kurgi short \| KEN \| S \| R \| S \| \| FR 48* \| 9745 \| Bhana 2 \| SDN \| R \| R \| R \| \| FR 49 \| 9830 \| Magawim buda 2 \| SDN \| S \| R \| S \| \| FR 50* \| 10302 \| Katingu \| THA \| R \| R \| S \| \| FR 51* \| 10757 \| No. 733 R; Oua kal \| TCD \| R \| - \| S \| \| FR 52* \| 10867 \| No. 1559; Selection bergere \| TCD \| R \| - \| S \| \| FR 53* \| 10969 \|  \| USA \| R \| S \| S \| \| FR 54* \| 11026 \| Col. No. 25 B; PI 329268 \| ETH \| R \| S \| S \| \| FR 55* \| 11374 \| Col. No. 772 B; PI 329629 \| ETH \| R \| - \| R \| \| FR 56 \| 11473 \| Col. No. R-109; PI 329738 \| ETH \| R \| S \| S \| \| FR 57* \| 11619 \| Col. No. R-330; PI 329897 \| ETH \| R \| R \| S \| \| FR 58* \| 11919 \| Col. No. P-83; PI 330201 \| ETH \| R \| R \| S \| \| FR 59 \| 12302 \| PI 287646 \| ZWE \| R \| S \| R \| \| FR 60 \| 12447 \| *S. bicolor* \| SDN \| R \| S \| S \| \| FR 61 \| 12697 \| PI 302116 \| AUS \| R \| S \| S \| \| FR 62 \| 12706 \| PI 2363; Orange; 161; *S. bicolor* \| USA \| R \| S \| S \| \| FR 63 \| 12735 \| PI 81216; *S. bicolor*; Migna \| YEM \| S \| S \| S \| \| FR 64 \| 12804 \| PI 170799; Akdari \| TUR \| S \| S \| R \| \| FR 65 \| 12883 \| PI 181077; Depar \| IND \| S \| S \| R \| \| FR 66 \| 12937 \| PI 196058 \| ETH \| S \| S \| S \| \| FR 67 \| 12945 \| PI 197050; MN 3107 \| NIC \| R \| S \| S \| \| FR 68 \| 12965 \| PI 208770 \| CUB \| R \| R \| R \| \| FR 69 \| 13264 \| PI 271619; *S. bicolor* \| IND \| S \| S \| R \| \| FR 70* \| 13294 \| PI 274509; *S. bicolor* \| VEN \| R \| R \| S \| \| FR 71* \| 13549 \| PI 291076 *S. bicolor* \| MEX \| R \| R \| R \| \| FR 72 \| 13782 \| PI 308211 *S. bicolor* \| ZAF \| R \| R \| S \| \| FR 73* \| 13893 \| PI 308340 *S. bicolor* \| ZAF \| S \| R \| S \| \| FR 74 \| 13919 \| PI 308368 *S. bicolor* \| ZAF \| R \| R \| S \| \| FR 75 \| 13971 \| PI 308427 *S. bicolor* \| ZAF \| R \| R \| S \| \| FR 76 \| 14010 \| PI 308488 *S. bicolor* \| ZAF \| R \| R \| S \| \| FR 77 \| 14090 \| PI 257292 \| ARG \| R \| R \| S \| \| FR 78 \| 14290 \|  \| BWA \| R \| S \| S \| \| FR 79* \| 14779 \| 1-1-3-2 \| CMR \| R \| R \| S \| \| FR 80* \| 14861 \| 1-2-4-6 \| CMR \| R \| S \| S \| \| FR 81* \| 15170 \| 2-4-6-3 \| CMR \| S \| R \| R \| \| FR 82* \| 15466 \| 2-2-2-9 \| CMR \| S \| - \| S \| \| FR 83* \| 15478 \| 2-2-6-7 \| CMR \| S \| R \| R \| \| FR 84* \| 15744 \| 4-1-7-3 \| CMR \| S \| S \| S \| \| FR 85* \| 15931 \| 4-2-20-1 \| CMR \| R \| R \| S \| \| FR 86* \| 15945 \| 4-3-2-5 \| CMR \| S \| R \| R \| \| FR 87* \| 16382 \| 2-2-9-2-A \| CMR \| S \| R \| S \| \| FR 88* \| 16528 \| 4-3-7-4 \| CMR \| R \| R \| R \| \| FR 89 \| 17941(SAR503) \|  \| IND \| R \| R \| S \| \| FR 90* \| 18039 \| Bhiwapur wani \| IND \| S \| R \| S \| \| FR 91* \| 19153 \| WM 10316 \| SDN \| S \| R \| S \| \| FR 92 \| 19389 \| Bangaladesh \| BGD \| R \| S \| S \| \| FR 93 \| 19445 \| Mahibidu \| BWA \| R \| R \| S \| \| FR 94 \| 19450 \| Tshabat sie-tshobat \| BWA \| R \| R \| S \| \| FR 95* \| 19859(KEP424) \| Badmasia \| IND \| S \| R \| S \| \| FR 96* \| 19975 \| SG 1730; Bassi \| SEN \| S \| S \| S \| \| FR 97* \| 20195 \| SG 2118; Karkaras \| NER \| R \| R \| S \| \| FR 98* \| 20298 \| SG 2182; Takanda \| NER \| R \| R \| S \| \| FR 99* \| 20625 \| HDW 749 \| USA \| R \| S \| R \| \| FR 100* \| 20632 \| HDW 736 \| USA \| R \| R \| R \| \| FR 101 \| 20635 \| HDW 351 \| USA \| R \| S \| - \| \| FR 102* \| 20679 \| HDW 325 \| USA \| R \| R \| S \| \| FR 103 \| 20697 \| HDW 418 \| USA \| S \| S \| S \| \| FR 104* \| 20713 \| HDW 330 \| USA \| R \| R \| S \| \| FR 105* \| 20740 \| HDW 518 \| USA \| S \| R \| S \| \| FR 106 \| 20747 \| HDW 11 \| USA \| R \| S \| S \| \| FR 107* \| 20762 \| HDW 540 \| USA \| R \| R \| S \| \| FR 108* \| 20767 \| HDW 147 \| USA \| S \| S \| S \| \| FR 109* \| 20771 \| HDW 544 \| USA \| R \| R \| S \| \| FR 110 \| 20777 \| HDW 505 \| USA \| R \| S \| - \| \| FR 111 \| 20816 \|  \| USA \| S \| R \| S \| \| FR 112* \| 20956 \| HDW 501 \| IND \| R \| R \| S \| \| FR 113* \| 21083 \| N0. 48928; Jogoo \| KEN \| R \| - \| R \| \| FR 114* \| 21425 \| Tengaramanga \| MWI \| S \| S \| - \| \| FR 115* \| 21512 \| Kashonthe \| MWI \| R \| S \| S \| \| FR 116 \| 21863 \| PI 253986 \| SYR \| S \| S \| S \| \| FR 117 \| 21891 \| BMR 8 \| USA \| S \| S \| S \| \| FR 118* \| 21897 \| HD 496 \| USA \| S \| - \| S \| \| FR 119 \| 22239(PMC15) \| Monoane \| BWA \| R \| S \| S \| \| FR 120 \| 22294(PMK108) \|  \| BWA \| R \| S \| R \| \| FR 121* \| 22609(JM4137) \|  \| LKA \| R \| - \| S \| \| FR 122 \| 22616(JM4217A) \| Sorghum shwebo 10 \| MYA \| S \| S \| S \| \| FR 123* \| 22720(DRM36) \|  \| SOM \| S \| R \| R \| \| FR 124 \| 22799(DRD106) \|  \| SOM \| S \| R \| S \| \| FR 125* \| 22986 \| K98 B \| SDN \| R \| R \| S \| \| FR 126* \| 23216(ZM137) \| Mampenga \| ZMB \| R \| S \| R \| \| FR 127 \| 23514(PAB26) \| Uluwale \| ETH \| S \| R \| S \| \| FR 128 \| 23521(PAB34) \| Utedit \| ETH \| S \| R \| S \| \| FR 129* \| 23579(PAB98) \| Tungo \| ETH \| S \| R \| S \| \| FR 130* \| 23586(PAB108) \| Ganga \| ETH \| S \| R \| S \| \| FR 131* \| 23590(PAB112) \| Ganga \| ETH \| S \| R \| S \| \| FR 132* \| 23644(RC41) \| Ditin \| GMB \| S \| S \| S \| \| FR 133* \| 23684(RPM84-2) \| Mele \| MOZ \| S \| S \| S \| \| FR 134* \| 23891 \| ALK 122 \| YEM \| S \| R \| S \| \| FR 135 \| 23992 \| AR 233 \| YEM \| S \| R \| R \| \| FR 136* \| 24139 \| TLSC 315 \| TZA \| R \| R \| S \| \| FR 137* \| 24175 \| TLSC 353 \| TZA \| R \| R \| S \| \| FR 138* \| 24218 \| TLSC 398 \| TZA \| R \| R \| S \| \| FR 139 \| 24348(VRR563) \| Mani \| IND \| S \| R \| S \| \| FR 140 \| 24365(VRR726) \| Shallu \| IND \| S \| S \| S \| \| FR 141 \| 24453 \| 62130 \| ZAF \| S \| S \| R \| \| FR 142 \| 24462 \| 67379 \| ZAF \| R \| S \| R \| \| FR 143 \| 24463 \| 67387 \| ZAF \| R \| S \| R \| \| FR 144 \| 24492 \| 68748 \| ZAF \| S \| S \| S \| \| FR 145 \| 24503 \| 71316 \| ZAF \| R \| S \| S \| \| FR 146* \| 24939 \| No. 2072 \| ZMB \| S \| R \| S \| \| FR 147* \| 24953 \| No. 2360 \| ZMB \| S \| - \| S \| \| FR 148* \| 25089(DSA147) \|  \| GHA \| R \| R \| S \| \| FR 149* \| 25249 \| Acc 71899 \| ETH \| S \| R \| S \| \| FR 150* \| 25301 \| Acc. 72761 \| ETH \| S \| R \| S \| \| FR 151* \| 25548(PS55) \| Kibumburi \| RWA \| S \| R \| S \| \| FR 152* \| 25732 \| SG 4642; Gadiaba die \| MLI \| R \| S \| S \| \| FR 153* \| 25836 \| SG 4806; Gadiaba ba \| MLI \| R \| R \| S \| \| FR 154* \| 25910 \| SG 4918; Kende ble \| MLI \| S \| S \| S \| \| FR 155* \| 25981 \| SG 5002; Bimbiri \| MLI \| R \| R \| S \| \| FR 156* \| 25989 \| SG 5040; Kendie die \| MLI \| R \| S \| S \| \| FR 157* \| 26025 \| SG5093; Keninke ba \| MLI \| R \| S \| S \| \| FR 158* \| 26046 \| SG 5118; Seguetene \| MLI \| R \| S \| S \| \| FR 159* \| 26222 \| TOGO 141; Goalogouaga \| TGO \| R \| S \| R \| \| FR 160* \| 26484 \| SG 4109; Esse tenha \| BEN \| S \| R \| R \| \| FR 161 \| 26617 \| WS 60 \| MDG \| R \| S \| R \| \| FR 162 \| 26694 \| 1958; Kwazulu engini \| ZAF \| R \| S \| S \| \| FR 163 \| 26701 \| 1988; Kwazulu hlehlemi \| ZAF \| S \| S \| S \| \| FR 164 \| 26737 \| No. 918; 65648 \| ZAF \| R \| R \| S \| \| FR 165 \| 26749 \| 67537; Breytenbach rooi \| ZAF \| R \| R \| R \| \| FR 166* \| 27034 \| WM 11548 \| SDN \| R \| - \| S \| \| FR 167* \| 27557 \| SG 6462; Yala yapson \| BFA \| R \| S \| R \| \| FR 168* \| 27697(PCI 36) \| Kagboyoh \| SLE \| R \| - \| R \| \| FR 169 \| 27786 \| 427 \| MAR \| S \| S \| S \| \| FR 170* \| 27887(JM4621) \|  \| YEM \| S \| S \| S \| \| FR 171* \| 28141 \| YSC 1402; PI 474787 \| YEM \| S \| S \| S \| \| FR 172* \| 28313 \| YSC 1675; PI 474972 \| YEM \| R \| S \| S \| \| FR 173 \| 28389 \| YSC 1773; PI 475052 \| YEM \| S \| S \| S \| \| FR 174 \| 28449 \| YSC 1850; PI 475116 \| YEM \| R \| R \| S \| \| FR 175 \| 28451 \| YSC 1853; PI 475119 \| YEM \| S \| R \| S \| \| FR 176 \| 28614 \| YSC 2091; PI 475292 \| YEM \| S \| S \| S \| \| FR 177* \| 28747 \| YSC 2404; PI 475429 \| YEM \| S \| S \| S \| \| FR 178* \| 28849 \| YSC 2695; PI 475552 \| YEM \| S \| R \| S \| \| FR 179* \| 29091(PHM104) \| Baida \| YEM \| S \| R \| S \| \| FR 180* \| 29100(PHM113) \| Qubali \| YEM \| S \| R \| S \| \| FR 181 \| 29187(PL14) \|  \| SWZ \| S \| S \| S \| \| FR 182 \| 29233(PL61) \|  \| SWZ \| R \| R \| S \| \| FR 183* \| 29239(PL67) \|  \| SWZ \| R \| R \| R \| \| FR 184 \| 29241(PL69) \|  \| SWZ \| S \| R \| S \| \| FR 185 \| 29269(PL104) \|  \| SWZ \| R \| S \| S \| \| FR 186 \| 29304(PL140) \|  \| SWZ \| R \| S \| S \| \| FR 187 \| 29314(PL152) \|  \| SWZ \| R \| S \| R \| \| FR 188 \| 29326(PL164) \|  \| SWZ \| R \| S \| S \| \| FR 189 \| 29335(PL173) \|  \| SWZ \| S \| S \| S \| \| FR 190 \| 29358(PHN2) \|  \| LSO \| R \| R \| R \| \| FR 191 \| 29392(PHM36) \|  \| LSO \| R \| R \| R \| \| FR 192 \| 29441(PC86) \|  \| LSO \| R \| S \| S \| \| FR 193 \| 29468(PC113) \|  \| LSO \| R \| S \| S \| \| FR 194 \| 29519(PC221) \|  \| LSO \| S \| R \| S \| \| FR 195 \| 29565(PHN139) \|  \| LSO \| R \| R \| S \| \| FR 196 \| 29568(PHN142) \|  \| LSO \| R \| R \| S \| \| FR 197 \| 29582(PHN160) \|  \| LSO \| R \| R \| S \| \| FR 198 \| 29606 \| Arnold 1757 \| ZAF \| S \| R \| R \| \| FR 199 \| 29627 \| Arnold 2457 \| ZAF \| S \| R \| R \| \| FR 200 \| 29654 \| Hong liuzi \| CHN \| S \| S \| R \| \| FR 201 \| 29689(AMM96-2) \|  \| ZWE \| R \| S \| S \| \| FR 202 \| 29714(AMM156) \|  \| ZWE \| R \| S \| S \| \| FR 203 \| 29733(AMM206) \|  \| ZWE \| R \| R \| S \| \| FR 204* \| 29772(AMM282) \|  \| ZWE \| R \| S \| S \| \| FR 205* \| 29914(AMM589) \|  \| ZWE \| S \| R \| S \| \| FR 206* \| 29950(AMM673) \|  \| ZWE \| R \| S \| S \| \| FR 207* \| 30079(AMM908) \|  \| ZWE \| S \| R \| S \| \| FR 208 \| 30092(AMM938) \|  \| ZWE \| R \| S \| R \| \| FR 209 \| 30231(AMM1314) \|  \| ZWE \| S \| S \| S \| \| FR 210 \| 30383 \| Xiao huang luo su jiao \| CHN \| S \| S \| R \| \| FR 211 \| 30400 \| Fanglan \| CHN \| S \| S \| S \| \| FR 212 \| 30417 \| Tie Sha mao \| CHN \| S \| S \| S \| \| FR 213 \| 30443 \| Houng ke er jiao \| CHN \| S \| S \| R \| \| FR 214 \| 30450 \| Lang wei ba \| CHN \| S \| R \| S \| \| FR 215 \| 30451 \| Jin ton gaoliang \| CHN \| S \| R \| S \| \| FR 216 \| 30460 \| Er fang lan \| CHN \| S \| S \| S \| \| FR 217 \| 30466 \| Gaoliang \| CHN \| S \| R \| S \| \| FR 218 \| 30507 \| SOG 102 \| KOR \| R \| S \| S \| \| FR 219 \| 30508 \| SOG 103 \| KOR \| R \| S \| S \| \| FR 220 \| 30533 \| SOG 129 \| KOR \| R \| S \| S \| \| FR 221 \| 30536 \| SOG 132 \| KOR \| R \| S \| S \| \| FR 222 \| 30562 \| SOG 159 \| KOR \| S \| R \| R \| \| FR 223* \| 30572(AD 12) \|  \| CMR \| R \| R \| R \| \| FR 224* \| 30838(AD572) \|  \| CMR \| S \| S \| S \| \| FR 225* \| 30986 \| US 110; Abako \| UGA \| R \| S \| S \| \| FR 226* \| 31043 \| US 171; Goda \| UGA \| R \| R \| S \| \| FR 227* \| 31172 \| US 317; Okwaras \| UGA \| R \| - \| S \| \| FR 228* \| 31186 \| US 331; Holish \| UGA \| R \| - \| S \| \| FR 229* \| 31299 \| US 450; Ntuku \| UGA \| S \| - \| S \| \| FR 230* \| 31485 \| US 642; Magune \| UGA \| R \| - \| S \| \| FR 231* \| 31557 \| IZ 82 \| BDI \| R \| R \| R \| \| FR 232* \| 31651 \| IZ 223; Amasaka \| ZAR \| S \| R \| S \| \| FR 233 \| 31681 \| *S. bicolor* 15; Beshna \| DZA \| S \| S \| S \| \| FR 234* \| 31706 \| YSC 727 \| YEM \| S \| R \| S \| \| FR 235* \| 31714 \| YSC 1032 \| YEM \| R \| R \| R \| \| FR 236* \| 32245 \| YSC 3521 \| YEM \| R \| S \| S \| \| FR 237 \| 32295 \| PC 81 \| IND \| S \| S \| S \| \| FR 238* \| 32349(AKG70) \|  \| IND \| S \| R \| S \| \| FR 239* \| 32439(AKG228) \| Kassava \| IND \| S \| S \| S \| \| FR 240* \| 32482(AKG313) \|  \| IND \| R \| R \| S \| \| FR 241* \| 32787 \| SOM 31 \| SOM \| S \| S \| S \| \| FR 242* \| 33023(AMF469) \|  \| TZA \| R \| S \| S \| \| FR 243* \| 33090 \| Criollo peloton \| HND \| R \| S \| S \| \| FR 244* \| 33353 \| ADS 22 \| KEN \| S \| S \| S \| \| FR 245 \| 33844(GSS194) \| Maldandi \| IND \| S \| R \| - \| \|  \| \| \| \| \| \| \| \| * Photosensitive accessions (122) in the minicore germplasm \| \| \| \| \| \| \| \|  \| \| \| \| \| \| \|   S- Susceptible & R - Resistant |
| --- | --- | --- | --- | --- | --- | --- | --- | --- | --- | --- | --- | --- | --- | --- | --- | --- | --- | --- | --- | --- | --- | --- | --- | --- | --- | --- | --- | --- | --- | --- | --- | --- | --- | --- | --- | --- | --- | --- | --- | --- | --- | --- | --- | --- | --- | --- | --- | --- | --- | --- | --- | --- | --- | --- | --- | --- | --- | --- | --- | --- | --- | --- | --- | --- | --- | --- | --- | --- | --- | --- | --- | --- | --- | --- | --- | --- | --- | --- | --- | --- | --- | --- | --- | --- | --- | --- | --- | --- | --- | --- | --- | --- | --- | --- | --- | --- | --- | --- | --- | --- | --- | --- | --- | --- | --- | --- | --- | --- | --- | --- | --- | --- | --- | --- | --- | --- | --- | --- | --- | --- | --- | --- | --- | --- | --- | --- | --- | --- | --- | --- | --- | --- | --- | --- | --- | --- | --- | --- | --- | --- | --- | --- | --- | --- | --- | --- | --- | --- | --- | --- | --- | --- | --- | --- | --- | --- | --- | --- | --- | --- | --- | --- | --- | --- | --- | --- | --- | --- | --- | --- | --- | --- | --- | --- | --- | --- | --- | --- | --- | --- | --- | --- | --- | --- | --- | --- | --- | --- | --- | --- | --- | --- | --- | --- | --- | --- | --- | --- | --- | --- | --- | --- | --- | --- | --- | --- | --- | --- | --- | --- | --- | --- | --- | --- | --- | --- | --- | --- | --- | --- | --- | --- | --- | --- | --- | --- | --- | --- | --- | --- | --- | --- | --- | --- | --- | --- | --- | --- | --- | --- | --- | --- | --- | --- | --- | --- | --- | --- | --- | --- | --- | --- | --- | --- | --- | --- | --- | --- | --- | --- | --- | --- | --- | --- | --- | --- | --- | --- | --- | --- | --- | --- | --- | --- | --- | --- | --- | --- | --- | --- | --- | --- | --- | --- | --- | --- | --- | --- | --- | --- | --- | --- | --- | --- | --- | --- | --- | --- | --- | --- | --- | --- | --- | --- | --- | --- | --- | --- | --- | --- | --- | --- | --- | --- | --- | --- | --- | --- | --- | --- | --- | --- | --- | --- | --- | --- | --- | --- | --- | --- | --- | --- | --- | --- | --- | --- | --- | --- | --- | --- | --- | --- | --- | --- | --- | --- | --- | --- | --- | --- | --- | --- | --- | --- | --- | --- | --- | --- | --- | --- | --- | --- | --- | --- | --- | --- | --- | --- | --- | --- | --- | --- | --- | --- | --- | --- | --- | --- | --- | --- | --- | --- | --- | --- | --- | --- | --- | --- | --- | --- | --- | --- | --- | --- | --- | --- | --- | --- | --- | --- | --- | --- | --- | --- | --- | --- | --- | --- | --- | --- | --- | --- | --- | --- | --- | --- | --- | --- | --- | --- | --- | --- | --- | --- | --- | --- | --- | --- | --- | --- | --- | --- | --- | --- | --- | --- | --- | --- | --- | --- | --- | --- | --- | --- | --- | --- | --- | --- | --- | --- | --- | --- | --- | --- | --- | --- | --- | --- | --- | --- | --- | --- | --- | --- | --- | --- | --- | --- | --- | --- | --- | --- | --- | --- | --- | --- | --- | --- | --- | --- | --- | --- | --- | --- | --- | --- | --- | --- | --- | --- | --- | --- | --- | --- | --- | --- | --- | --- | --- | --- | --- | --- | --- | --- | --- | --- | --- | --- | --- | --- | --- | --- | --- | --- | --- | --- | --- | --- | --- | --- | --- | --- | --- | --- | --- | --- | --- | --- | --- | --- | --- | --- | --- | --- | --- | --- | --- | --- | --- | --- | --- | --- | --- | --- | --- | --- | --- | --- | --- | --- | --- | --- | --- | --- | --- | --- | --- | --- | --- | --- | --- | --- | --- | --- | --- | --- | --- | --- | --- | --- | --- | --- | --- | --- | --- | --- | --- | --- | --- | --- | --- | --- | --- | --- | --- | --- | --- | --- | --- | --- | --- | --- | --- | --- | --- | --- | --- | --- | --- | --- | --- | --- | --- | --- | --- | --- | --- | --- | --- | --- | --- | --- | --- | --- | --- | --- | --- | --- | --- | --- | --- | --- | --- | --- | --- | --- | --- | --- | --- | --- | --- | --- | --- | --- | --- | --- | --- | --- | --- | --- | --- | --- | --- | --- | --- | --- | --- | --- | --- | --- | --- | --- | --- | --- | --- | --- | --- | --- | --- | --- | --- | --- | --- | --- | --- | --- | --- | --- | --- | --- | --- | --- | --- | --- | --- | --- | --- | --- | --- | --- | --- | --- | --- | --- | --- | --- | --- | --- | --- | --- | --- | --- | --- | --- | --- | --- | --- | --- | --- | --- | --- | --- | --- | --- | --- | --- | --- | --- | --- | --- | --- | --- | --- | --- | --- | --- | --- | --- | --- | --- | --- | --- | --- | --- | --- | --- | --- | --- | --- | --- | --- | --- | --- | --- | --- | --- | --- | --- | --- | --- | --- | --- | --- | --- | --- | --- | --- | --- | --- | --- | --- | --- | --- | --- | --- | --- | --- | --- | --- | --- | --- | --- | --- | --- | --- | --- | --- | --- | --- | --- | --- | --- | --- | --- | --- | --- | --- | --- | --- | --- | --- | --- | --- | --- | --- | --- | --- | --- | --- | --- | --- | --- | --- | --- | --- | --- | --- | --- | --- | --- | --- | --- | --- | --- | --- | --- | --- | --- | --- | --- | --- | --- | --- | --- | --- | --- | --- | --- | --- | --- | --- | --- | --- | --- | --- | --- | --- | --- | --- | --- | --- | --- | --- | --- | --- | --- | --- | --- | --- | --- | --- | --- | --- | --- | --- | --- | --- | --- | --- | --- | --- | --- | --- | --- | --- | --- | --- | --- | --- | --- | --- | --- | --- | --- | --- | --- | --- | --- | --- | --- | --- | --- | --- | --- | --- | --- | --- | --- | --- | --- | --- | --- | --- | --- | --- | --- | --- | --- | --- | --- | --- | --- | --- | --- | --- | --- | --- | --- | --- | --- | --- | --- | --- | --- | --- | --- | --- | --- | --- | --- | --- | --- | --- | --- | --- | --- | --- | --- | --- | --- | --- | --- | --- | --- | --- | --- | --- | --- | --- | --- | --- | --- | --- | --- | --- | --- | --- | --- | --- | --- | --- | --- | --- | --- | --- | --- | --- | --- | --- | --- | --- | --- | --- | --- | --- | --- | --- | --- | --- | --- | --- | --- | --- | --- | --- | --- | --- | --- | --- | --- | --- | --- | --- | --- | --- | --- | --- | --- | --- | --- | --- | --- | --- | --- | --- | --- | --- | --- | --- | --- | --- | --- | --- | --- | --- | --- | --- | --- | --- | --- | --- | --- | --- | --- | --- | --- | --- | --- | --- | --- | --- | --- | --- | --- | --- | --- | --- | --- | --- | --- | --- | --- | --- | --- | --- | --- | --- | --- | --- | --- | --- | --- | --- | --- | --- | --- | --- | --- | --- | --- | --- | --- | --- | --- | --- | --- | --- | --- | --- | --- | --- | --- | --- | --- | --- | --- | --- | --- | --- | --- | --- | --- | --- | --- | --- | --- | --- | --- | --- | --- | --- | --- | --- | --- | --- | --- | --- | --- | --- | --- | --- | --- | --- | --- | --- | --- | --- | --- | --- | --- | --- | --- | --- | --- | --- | --- | --- | --- | --- | --- | --- | --- | --- | --- | --- | --- | --- | --- | --- | --- | --- | --- | --- | --- | --- | --- | --- | --- | --- | --- | --- | --- | --- | --- | --- | --- | --- | --- | --- | --- | --- | --- | --- | --- | --- | --- | --- | --- | --- | --- | --- | --- | --- | --- | --- | --- | --- | --- | --- | --- | --- | --- | --- | --- | --- | --- | --- | --- | --- | --- | --- | --- | --- | --- | --- | --- | --- | --- | --- | --- | --- | --- | --- | --- | --- | --- | --- | --- | --- | --- | --- | --- | --- | --- | --- | --- | --- | --- | --- | --- | --- | --- | --- | --- | --- | --- | --- | --- | --- | --- | --- | --- | --- | --- | --- | --- | --- | --- | --- | --- | --- | --- | --- | --- | --- | --- | --- | --- | --- | --- | --- | --- | --- | --- | --- | --- | --- | --- | --- | --- | --- | --- | --- | --- | --- | --- | --- | --- | --- | --- | --- | --- | --- | --- | --- | --- | --- | --- | --- | --- | --- | --- | --- | --- | --- | --- | --- | --- | --- | --- | --- | --- | --- | --- | --- | --- | --- | --- | --- | --- | --- | --- | --- | --- | --- | --- | --- | --- | --- | --- | --- | --- | --- | --- | --- | --- | --- | --- | --- | --- | --- | --- | --- | --- | --- | --- | --- | --- | --- | --- | --- | --- | --- | --- | --- | --- | --- | --- | --- | --- | --- | --- | --- | --- | --- | --- | --- | --- | --- | --- | --- | --- | --- | --- | --- | --- | --- | --- | --- | --- | --- | --- | --- | --- | --- | --- | --- | --- | --- | --- | --- | --- | --- | --- | --- | --- | --- | --- | --- | --- | --- | --- | --- | --- | --- | --- | --- | --- | --- | --- | --- | --- | --- | --- | --- | --- | --- | --- | --- | --- | --- | --- | --- | --- | --- | --- | --- | --- | --- | --- | --- | --- | --- | --- | --- | --- | --- | --- | --- | --- | --- | --- | --- | --- | --- | --- | --- | --- | --- | --- | --- | --- | --- | --- | --- | --- | --- | --- | --- | --- | --- | --- | --- | --- | --- | --- | --- | --- | --- | --- | --- | --- | --- | --- | --- | --- | --- | --- | --- | --- | --- | --- | --- | --- | --- | --- | --- | --- | --- | --- | --- | --- | --- | --- | --- | --- | --- | --- | --- | --- | --- | --- | --- | --- | --- | --- | --- | --- | --- | --- | --- | --- | --- | --- | --- | --- | --- | --- | --- | --- | --- | --- | --- | --- | --- | --- | --- | --- | --- | --- | --- | --- | --- | --- | --- | --- | --- | --- | --- | --- | --- | --- | --- | --- | --- | --- | --- | --- | --- | --- | --- | --- | --- | --- | --- | --- | --- | --- | --- | --- | --- | --- | --- | --- | --- | --- | --- | --- | --- | --- | --- | --- | --- | --- | --- | --- | --- | --- | --- | --- | --- | --- | --- | --- | --- | --- | --- | --- | --- | --- | --- | --- | --- | --- | --- | --- | --- | --- | --- | --- | --- | --- | --- | --- | --- | --- | --- | --- | --- | --- | --- | --- | --- | --- | --- | --- | --- | --- | --- | --- | --- | --- | --- | --- | --- | --- | --- | --- | --- | --- | --- | --- | --- | --- | --- | --- | --- | --- | --- | --- | --- | --- | --- | --- | --- | --- | --- | --- | --- | --- | --- | --- | --- | --- | --- | --- | --- | --- | --- | --- | --- | --- | --- | --- | --- | --- | --- | --- | --- | --- | --- | --- | --- | --- | --- | --- | --- | --- | --- | --- | --- | --- | --- | --- | --- | --- | --- | --- | --- | --- | --- | --- | --- | --- | --- | --- | --- | --- | --- | --- | --- | --- | --- | --- | --- | --- | --- | --- | --- | --- | --- | --- | --- | --- | --- | --- | --- | --- | --- | --- | --- | --- | --- | --- | --- | --- | --- | --- | --- | --- | --- | --- | --- | --- | --- | --- | --- | --- | --- | --- | --- | --- | --- | --- | --- | --- | --- | --- | --- | --- | --- | --- | --- | --- | --- | --- | --- | --- | --- | --- | --- | --- | --- | --- | --- | --- | --- | --- | --- | --- | --- | --- | --- | --- | --- | --- | --- | --- | --- | --- | --- | --- | --- | --- | --- | --- | --- | --- | --- | --- | --- | --- | --- | --- | --- | --- | --- | --- | --- | --- | --- | --- | --- | --- | --- | --- | --- |
